# Best evidence summary on positioning management in stroke patients

**DOI:** 10.3389/fneur.2025.1648841

**Published:** 2025-10-30

**Authors:** Yuanfang Xiong, Mingxia Pan, Wenting Chai, Huijuan Lei, Huan Peng, Ziping Hu, Na Li, Yongqi Liang, Lingyu Kuang, Hanjiao Liu

**Affiliations:** ^1^Department of Nursing, Shenzhen Hospital of Integrated Traditional Chinese and Western Medicine, Shenzhen, China; ^2^School of Nursing, Fujian University of Traditional Chinese Medicine, Fuzhou, China; ^3^School of Nursing, Guangdong Pharmaceutical University, Guangzhou, China; ^4^School of Nursing, Guangzhou University of Chinese Medicine, Guangzhou, China

**Keywords:** stroke, position, evidence-based nursing, summary of evidence, management

## Abstract

**Background:**

Stroke is the third leading cause of death in the world, characterized by high morbidity, high mortality, high disability and high recurrence rates, which brings a heavy burden to families and society. The implementation of positioning management for stroke patients can effectively improve their clinical outcomes and quality of life; however, the current evidence related to stroke is fragmented, which is not conducive to its utilization by clinical healthcare professionals.

**Objective:**

A systematic retrieval, critical appraisal, and synthesis of evidence on positioning management strategies for stroke patients were conducted to establish an evidence-based foundation for clinical decision-making in neurological rehabilitation.

**Methods:**

Based on the “6S” evidence resource pyramid, a top-down search strategy was employed, searching relevant databases and guideline websites, including the Scottish Intercollegiate Guidelines Network, National Institute of Health and Care Excellence, the American Heart Association, Cochrane Library, Embase, PubMed, Web of Science, CINAHL, CNKL, VIP, the Wanfang database, China Biology Medicine, UpToDate, Chinese Medical Association, the Yi Maitong Guidelines Network, Dingxiangyuan. Various types of literature such as clinical guidelines, expert consensus, systematic reviews, meta-analyses, and evidence summaries were also included. The search period covered February 2015 to February 2025. Two reviewers independently screened and critically assessed the literature, and then extracted and synthesized the evidence by grading it according to the Joanna Briggs Institute Centre for Evidence-Based Health Care Evidence Pre-grading System, Australia (2016 version).

**Results:**

A total of 9,605 publications were retrieved, resulting in the inclusion of 12 publications, including nine clinical guidelines, one clinical decision support tool, one systematic review, and one expert consensuses. The evidence was synthesized into seven thematic areas: team composition, comprehensive assessment, head-of-bed elevation angle, body positioning Strategies, early mobilization, assistive devices, and clinical considerations. Resulting in 37 evidence-based practice recommendations.

**Conclusion:**

This study summarizes the best evidence for positional management of stroke patients, which provides an evidence-based basis for standardizing stroke positional management. However, the best evidence should be used in an individualized manner with comprehensive consideration of the actual clinical situation when the evidence is applied in order to improve the clinical outcomes and quality of life of stroke patients. In the future, it should also be combined with multi-sample and multi-center studies to validate its effect, as well as to further enrich the content of stroke position management.

## 1 Introduction

Stroke is the third leading cause of death globally, presenting a quadruple burden of high morbidity, disability, mortality and risk of recurrence. In 2021, there were 93.8 million stroke survivors and 11.9 million new stroke cases worldwide. There are 7.3 million global stroke deaths, accounting for 10.7% of all deaths (1). Motor impairment is one of the main disabilities associated with stroke (2). Movement disorders include weakness, spasticity, abnormal motor coordination and motor control disorders (3). During the recovery process, the gradual restoration of muscle strength is frequently accompanied by the development of spasticity (4). Post-stroke spasticity is the most common complication of stroke. The study showed that the total prevalence of spasticity after stroke was 25.3%, and 26.7% after the first stroke (5). Spasticity and its associated abnormal joint postures often interact with weakness and loss of dexterity, leading to dysfunctional motor control and functional limitations that can severely impact a patient’s ability to live and participate in society (6).

Patient positioning constitutes a fundamental nursing intervention (7). Notably, across diverse clinical scenarios, specific body positions can be utilized to confer therapeutic benefits for selected patients. In other words, under certain circumstances, goal-oriented therapeutic positions may take precedence over routine positioning, as they facilitate the improvement of patients’ physiological functions while promoting recovery (8).

In the application of positioning management for stroke patients, beyond its direct impact on spasticity, the clinical significance of postural changes further manifests in the regulation of vital signs during the acute phase (9). For stroke survivors, maintaining improper postures over an extended period can exacerbate spasticity in the affected limbs. This not only increases the risk of developing problems such as shoulder subluxation, shoulder pain, joint external rotation, foot inversion, or foot drop but also hinders the restoration of muscular strength, functional recovery, standing ability and walking performance. In addition, in the acute phase of stroke, postural changes play a crucial role in modulating vital physical signs. Specifically, they have a significant impact on oxygenation status, systemic blood pressure regulation, and cerebrovascular dynamics, including cerebral perfusion pressure, arterial flow velocity, and the maintenance of intracranial pressure balance (10, 11). Meanwhile, appropriate postural change is also intricately linked to the recovery of limb motor function and the prevention of secondary complications (12). In conclusion, positioning management is of great significance for stroke patients and serves as a key part of clinical nursing.

Given these circumstances, it becomes evident that implementing evidence-based postural management is of great significance in stroke rehabilitation. Positioning management involves the deliberate adjustment of body alignment, positioning, and support methods. Its core goals are to prevent complications, enhance functional capabilities, and improve patient comfort and quality of life. By understanding these aspects, we can better explore how to develop more effective positioning management strategies, which is precisely the focus of this study.

Currently, there are guidelines and systematic reviews available regarding postural management for critically ill patients (13), surgical patients (14), and patients with acute respiratory distress syndrome (ARDS) (15), which hold significant guiding value for the clinical management of patient positioning. However, there is still a lack of standardized and actionable protocols for positional management of stroke patients. The available evidence is scattered across multiple, often inconsistent guidelines and systematic reviews, which hinders effective translation into clinical practice. To address this gap, our study systematically synthesizes the best available evidence on postural management after stroke through thorough literature retrieval and rigorous critical appraisal. This consolidation aims to develop an evidence-based framework that can effectively guide clinical decision-making and ultimately improve rehabilitation outcomes for stroke patients.

## 2 Methodology

### 2.1 Establishment of evidence-based questions

This study utilized the PIPOST model, developed by the JBI Center for Evidence-Based Nursing at Fudan University in Shanghai, as the theoretical framework for the analysis. The PIPOST framework encompasses the following components: (i) P (Population): The target population consists of stroke patients, for whom evidence-based interventions are being applied. (ii) I (Intervention): This refers to studies focusing on postural management interventions aimed at improving patient outcomes. (iii) P (Professional): The professionals involved in the implementation of these interventions include nurses, physicians, rehabilitation specialists, and other healthcare providers. (iv) O (Outcome): The outcomes assessed in this study include changes in limb function, quality of life, and the incidence of complications related to postural management in stroke patients. (v) S (Setting): The settings for the implementation of interventions include inpatient wards, rehabilitation centers, and community-based environments. (vi) T (Type of Evidence): Eligible evidence types include clinical practice guidelines, expert consensus statements, practice recommendations, clinical decision-making protocols, evidence summaries, and systematic reviews.

### 2.2 Evidence retrieval

The following databases and websites were searched.

The following Chinese databases were used: China Knowledge Resource Integrated database (CNKI), Wanfang database, and VIP database.

The following English databases were used: PubMed, Embase, Web of Science, Cochrane Library, and CINAHL.

Guidelines networks: the Scottish Intercollegiate Guidelines Network (SIGN), Ding Xiangyuan, YI Maitong, National Institute of Health and Clinical Excellence (NICE), UpToDate, American Heart Association/American Stroke Association (AHA/ASA), and Chinese Medical Association.

Search strategy. Since there are no specific guidelines or expert consensus on positioning management, and relevant content is scattered in the guidelines and expert consensus, to ensure the accuracy of the search, we used different search styles according to the type of literature. The search formula was as follows: (i) (stroke* OR apoplexy OR “cerebral infarction” OR “cerebral hemorrhage” OR “cerebrovascular accident*” OR “cerebrovascular stroke*” OR “brain vascular accident*” OR “neurological illness” OR “Cerebrovascular Accident” OR “cerebral infarctions”) AND (guideline∗ OR “practice guideline” OR routine∗ OR “recommended practice” OR consensus∗); (ii) (stroke* OR apoplexy OR “cerebral infarction” OR “cerebral hemorrhage” OR “cerebrovascular accident*” OR “cerebrovascular stroke*” OR “brain vascular accident*” OR rehabilitation OR “neurological illness” OR “Cerebrovascular Accident”) AND (“evidence summary” OR “systematic review” OR “meta-analysis”) AND (positioning OR spasticity OR “good limb position” OR rehabilitation). The search covered the period from the February 2015 to February 2025.

### 2.3 Inclusion and exclusion criteria

The inclusion criteria were as follows: (i) Studies focused on positioning management, spasticity management, and dyskinesia management in stroke patients, (ii) Studies including clinical guidelines, expert consensus statements, evidence summaries, and systematic reviews, (iii) Studies published in both Chinese and English languages.

Exclusion criteria were as follows: (i) Literature type was conference abstract, guideline interpretation, research plan/proposal or the old guide that has been replaced. (ii) Literatures with failed quality evaluation.

### 2.4 Literature screening

The literature was independently screened by two postgraduate students who had received specialized training in evidence-based nursing. The screening process followed a systematic approach, which included the following steps: (i) Deduplication: Duplicate entries were identified and removed using EndNote software, (ii) Initial Screening: The titles and abstracts of the articles were reviewed, and those not relevant to the research question were excluded, (iii) Rescreening: The remaining articles were subjected to a detailed review, and eligible studies were selected based on predefined inclusion criteria. Basic information was extracted from the selected studies, including the first author’s name, affiliation, publication year, source, evidence type, and article topic. To ensure accuracy, the screening results were cross-checked by both researchers. In cases of disagreement, a third expert in evidence-based nursing was consulted to resolve discrepancies.

### 2.5 Evaluation of the quality of the literature

The tool employed for the evaluation of the guidelines is the Assessment of Guidelines for Research and Evaluation II (AGREE II) (16), a widely recognized framework for assessing the methodological quality of clinical practice guidelines. The AGREE II tool evaluates guidelines across six key areas: scope and purpose, participants, rigor, clarity, applicability, and independence. Each of these areas is assessed using a scale from 1 to 7, where 1 represents strongly disagree and 7 represents strongly agree. The higher the degree to which the guidelines meet the specified criteria, the higher the corresponding score. The standardized percentage score for each item was calculated according to the following formula: (Actual Score−Lowest Possible Score) / (Highest Possible Score−Lowest Possible Score) × 100%. Items achieving a standardized percentage score of ≥60% were graded as A, those with a score between ≥30% and <60% were graded as B, and items with a score of <30% were graded as C. For the evaluation of systematic reviews, meta-analyses, and expert consensus, the Australian JBI Centre for Evidence-Based Health Care’s Quality Assessment Criteria (2016) (17) was applied. Evaluators provided judgments of “yes,” “no,” “unclear,” or “inapplicable” for each item based on a thorough review of the relevant literature. Following group discussions, consensus decisions were made regarding the inclusion, exclusion, or need for further clarification for any item rated as “no,” “unclear,” or “inapplicable.” At present, there is a lack of internationally recognized quality assessment tools specifically designed for the evaluation of clinical decision-making frameworks and evidence summaries. To address this methodological gap, the following approach was adopted: (i) Evidence derived from authoritative databases was classified *a priori* as high-quality, based on the credibility and reputation of the institution, (ii) For evidence obtained from alternative sources, a comprehensive full-text appraisal was conducted to ensure strict adherence to established evidence development protocols. The quality of the studies included in this analysis was independently assessed by two researchers, both of whom had undergone formal training in evidence-based methods. In instances of disagreement between the assessors, any discrepancies were resolved through collaborative discussion or by consulting a third specialist with expertise in evidence-based care.

### 2.6 Evidence extraction and summary

Studies that met the predefined inclusion and exclusion criteria were independently screened by two researchers with systematic evidence-based training and expertise in stroke care. This screening process involved reviewing titles, abstracts, and full texts of the studies. The researchers then extracted relevant data and basic information from the selected studies, cross-checking their results for consistency.

In cases of discrepancies, a consensus was reached through discussion with a third researcher to resolve any differences. When evidence from different sources was complementary or when conclusions were consistent, synthesis or generalization was employed. However, in instances where conflicting evidence emerged, the principles of evidence-based prioritization were applied, with preference given to high-quality evidence and the most recent authoritative publications.

Additionally, the synthesized evidence was graded using the Joanna Briggs Institute (JBI) methodology, as outlined by the JBI Center for Evidence-Based Medicine in Australia (18).

## 3 Results

### 3.1 Search results

**Figure 1:**
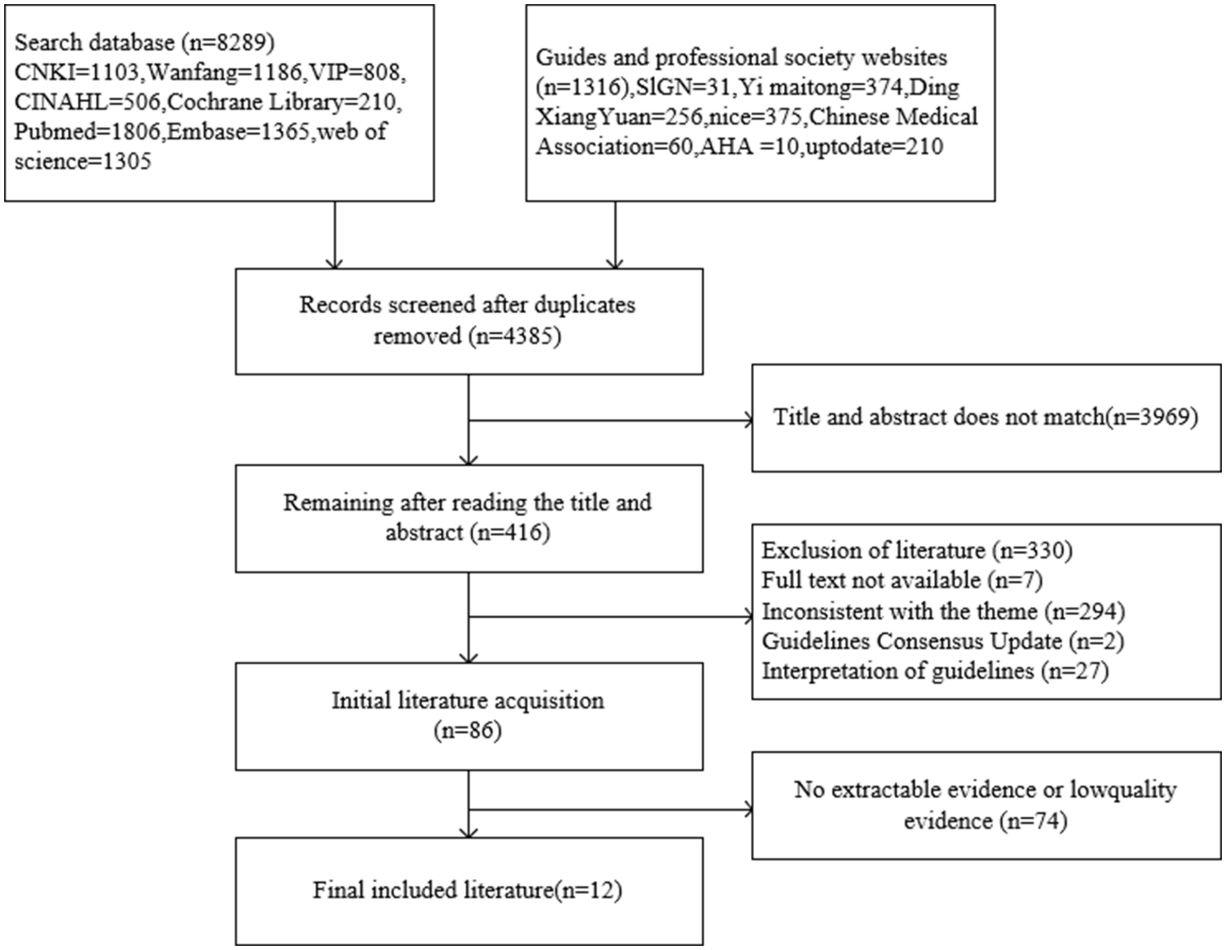
Flow diagram of literature search.

The initial database search identified a total of 9,605 articles. After removing duplicates, 4,385 articles remained for further review. Titles, abstracts, and full texts were systematically screened to exclude articles that did not meet the inclusion criteria. Following this process, a total of 12 articles were ultimately included in the study. These comprised nine clinical practice guidelines (19–27), one clinical decision-making article (28), one systematic review (29) and one expert consensus (30). The basic characteristics of the included articles are summarized in Table 1, and the detailed literature screening process is illustrated in Figure 1.

**Table 1:** The general characteristics of the included literature.

Included literatures	Year of publication	Literature sources	Type of literature	Title of literature
NICE (19)	2023	NICE	Guideline	Adult stroke rehabilitation
Zhang et al. (20)	2023	CNKI	Guideline	Cerebrovascular rehabilitation
Cai et al. (21)	2021	CNKI	Guideline	Stroke prevention and treatment guidelines
West China Center for Evidence-Based Nursing, Sichuan University, nursing management professional Committee of Chinese Nursing Association, neurosurgery branch of Chinese Medical Association (22)	2021	CNKI	Guideline	Enteral nutritional care for stroke
Robert et al. (23)	2019	Pubmed	Guideline	Stroke rehabilitation
Chinese Society of Neurology, Chinese Medical Association, Neurorehabilitation group, cerebrovascular disease group (24)	2017	CNKI	Guideline	Stroke rehabilitation
Yang et al. (25)	2016	CNKI	Guideline	Stroke nursing
Winstein et al. (26)	2016	AHA	Guideline	Adult stroke rehabilitation
Torbey et al. (27)	2015	Pubmed	Guideline	Management of cerebral infarction
Filho and Mullen (28)	2024	Uptodate	clinical decision	Management of cerebral infarction
Li et al. (29)	2015	CNKI	Systematic review	Temporal differences in positional placement
Bavikatte et al. (30)	2021	CINAHL	Expert consensus	Early recognition, intervention and management of post-stroke spasticity

NICE, National Institute of Health and Care Excellence. CNKI, China National Knowledge Infrastructure.

### 3.2 Results of the evaluation of the quality of the included studies

#### 3.2.1 Quality evaluation results of the guidelines

The guidelines were evaluated using AGREE II and the results are shown in Table 2, all with a recommendation level of A or B. The overall quality was high and inclusion was granted.

**Table 2:** Guide quality evaluation results.

Guidelines	Percentage of field standardization						≥60% field number (*n*)	≥30% field number (*n*)	Recommendation level
	Scope and purpose	Involved personnel	Preciseness of guidelines	Clarity of guideline	Applicability of guidelines	Independence of guidelines			
NICE (19)	68.51	72.22	84.02	90.74	79.16	83.33	6	6	A
Zhang et al. (20)	94.44	72.22	81.25	88.89	84.72	100	6	6	A
Cai et al. (21)	96.29	87.03	88.89	92.59	73.61	58.33	5	6	B
West China Center for Evidence-Based Nursing, Sichuan University, nursing management professional Committee of Chinese Nursing Association, neurosurgery branch of Chinese Medical Association (22)	94.44	97.22	88.54	94.44	68.75	62.50	6	6	A
Teasell et al. (23)	96.29	88.89	75.69	77.78	72.22	94.44	6	6	A
Chinese Society of Neurology, Chinese Medical Association, Neurorehabilitation group, cerebrovascular disease group (24)	74.07	55.56	55.56	55.6	56.94	52.78	1	6	B
Yang et al. (25)	100	68.52	75	88.89	56.94	50	4	6	B
Winstein et al. (26)	92.59	90.74	75	85.19	57.14	80.56	5	6	B
Torbey et al. (27)	88.89	72.22	63.89	72.22	63.89	94.44	6	6	A

#### 3.2.2 Systematic evaluation or meta-analysis quality evaluation results

One systematic review (29) was included in the study. The evaluation results can be found in Table 3.

**Table 3:** Meta-analysis quality evaluation result.

Inclusion of literature	①	②	③	④	⑤	⑥	⑦	⑧	⑨	⑩	⑪
Li et al. (29)	Yes	Yes	Yes	Yes	Yes	Yes	Yes	Yes	No	Yes	Yes

① Did the research questions and inclusion criteria for the review include the components of PICO? ② whether the literature inclusion criteria were appropriate for that evidence-based question; ③ whether the search strategy used was appropriate; ④ whether the sources of the research papers were appropriate; ⑤ whether the criteria used to evaluate the quality of the literature were appropriate; ⑥ whether the evaluation of the quality of the literature was done independently by two or more evaluators; ⑦ whether the data were extracted with measures to reduce errors; ⑧ whether the methods used to synthesize/combine studies were appropriate; ⑨ whether possible publication bias was assessed; ⑩ whether recommendations for policy and/or practice were made with the support of the reported data; and; ⑪ whether appropriate recommendations were made for future directions for further research.

#### 3.2.3 Quality evaluation results of the expert consensus

One expert consensuses (30) from CINAHL was included in the study. The details are shown in Table 4.

**Table 4:** Expert consensus quality evaluation result.

Inclusion of literature	①	②	③	④	⑤	⑥
Bavikatte et al. (30)	Yes	Yes	Yes	Yes	Yes	Yes

① Is the source of the opinion clearly identified? ② does the source of the viewpoint have standing in the field of expertise? ③ Are the interests of relevant groups the central focus of opinions? ④ Is the stated position the result of the analysis process? Is the viewpoint expressed logically? ⑤ Do you have any references to existing literature? and ⑥ Whether there is any inconsistency between the viewpoints presented and the previous literature.

### 3.3 Summary and description of evidence

The evidence was extracted from the final literature. Through the induction and integration of the evidence, the evidence was finally summarized from six domains: team composition, comprehensive assessment, head-of-bed elevation angle, body positioning Strategies, early mobilization, assistive devices, and clinical considerations and 37 best evidences were formed, providing a comprehensive framework for neurorehabilitation practice, as shown in Table 5.

**Table 5:** Results of literature extraction and grading of evidence.

Evidence items	Evidence content	Level of evidence
Team composition	1. It is recommended that stroke patients who are candidates for postacute rehabilitation receive organized, coordinated, interprofessional care (23).	1b
	2. Core members include: the core team of rehabilitation professionals should include physicians, physical therapists, therapists, nurses, patients and families (23).	1b
Comprehensive assessment	1. After a patient is admitted to the hospital, a preliminary assessment should be conducted by rehabilitation professionals as soon as possible, and it is preferable to complete the initial screening and assessment within 48 h (23).	1b
	2. The assessment includes: the patient’s vital signs, physical function, willingness to learn and ability to participate in rehabilitation (23).	5b
Head-of-bed elevation angle	1. In the acute phase, the presence of increased intracranial pressure and the risk of aspiration as well as cardiopulmonary disease should be taken into account, and the position and height of the head of the bed should be adjusted individually, avoiding drastic changes in position (28).	5b
	2. For patients in the acute phase of stroke who are at risk for any of the following problems, increased intracranial pressure, aspiration, cardiopulmonary decompensation, or decreased oxygen saturation keep the head in a neutral position and in line with the body and raise the head of the bed to 30 degrees. If there are no such problems, keep the head of the bed in the most comfortable position for the patient (21, 27, 28).	1b
	3. Most patients with cerebral infarction can be placed in a horizontal position (22, 27).	3e
	4. Raise the head of the bed ≥30° when administering enteral nutrition, if condition permits (22).	1a
	5. Continue to maintain the original position for more than 30 min after enteral nutrition (22).	3c
	6. If the condition permits, raise the head of the bed 15 ~ 30°, with the patient lying on their side and keeping the head slightly tilted back when sputum suctioning is required for patients undergoing enteral nutrition. Improve the tolerance level of sputum aspiration (22).	5b
Body positioning strategies	1. Stroke patients can be placed in the anti-spasticity position, which can effectively reduce limb spasticity and improve the functional rehabilitation efficacy of the affected side of the limb (20, 23).	1b
	2. Encourage lying on the affected side, appropriate lying on the healthy side. Use the supine position as little as possible, and try to avoid the semi-recumbent position. Maintain correct sitting and standing position (20).	5b
	3. When the patient’s vital signs are stable and neurological symptoms have not progressed for 48 h, the limb should be positioned in an antispastic position immediately upon hospital admission (25).	2a
	4. The position of post-stroke patients should be changed every 1–2 h without affecting the patient’s vital signs (25), and the duration of the antispasmodic position is recommended to be more than 4 weeks (29).	2b
	5. For shoulder-hand syndrome and edema, moderate elevation of the affected limb is recommended (20, 23).	2b
	6. Positioning of hemiplegic shoulder in maximum external rotation while the patient is either sitting or in bed for 30 min daily is probably indicated (26).	1c
	7. When lying on the healthy side, the affected side is on top, supported by a pillow in front of the body, with the affected side’s upper limb naturally extended and the affected side’s lower limb flexed (21, 25).	2a
	8. When lying on the affected side, the affected side is underneath, supported by a pillow behind the back. The affected side’s upper limb is extended, its lower limb slightly flexed; the healthy side’s upper limb is in a natural position, and its lower limb is in a stepping position (21, 25).	2a
	9. Supine position: the shoulder and hip of the affected side are supported by thin pillows, and the head is slightly turned to the affected side. The upper arm of the affected side is rotated and abducted 20°–40°, with its elbow and wrist joints straightened, fingers stretched out, and palm turned upward. The affected side’s knee is slightly cushioned, and its foot’s toes are turned upward (21).	2a
	10. Wheelchair sitting position: The patient’s back should be against the chair back, with the trunk straight and upper body slightly forward. The affected upper limb rests on a chest pillow, with fingers naturally extended. To correct external rotation of the affected foot, the hip, knee, and ankle joints should be flexed at 90°, the feet flat and perpendicular to the legs, toes pointing forward, and feet shoulder-width apart (21).	2a
	11. Bed sitting position: Support the patient’s back, shoulders, arms, and lower limbs with soft pillows, or raise the head of the bed to 90°. Keep the trunk straight without leaning forward, with elbow joints flexed at 90°, knees flexed at 50°–60° (supported by soft pillows under the knees), and a pillow under the patient’s feet to keep the soles supported. For the affected upper limbs, stretch them forward and place them straight on an overbed table or adjustable board, while keeping the knee joints extended (21, 25).	2a
	12. Moderate risk of pressure ulcers: turn them over at least once every 2 h, use wedge cushions, and maintain a 30° lateral position. When the head of the bed must be elevated more than 30° or placed in a semi-recumbent position due to the condition, first elevate the foot of the bed to a certain height, then raise the head of the bed. If unable to elevate the foot of the bed, place a support under the buttocks to decompress the area. Severe risk: Ensure turning frequency (every 2 h), increase the number of minor repositioning’s, use a wedge cushion, and maintain a 30° lateral position. Additionally, if the patient is in an anterior tilt, left/right lateral recline, or posterior tilt position, change the sitting position every 15 to 30 min (25).	2b
Early mobilization	1. In stable clinical condition, patients with mild to moderate stroke may start bedside and early out-of-bed activities 24 h after stroke onset, including transitioning from bedside sitting to upright position, bedside standing, bed-to-chair transfer and ambulation. These activities should be progressive and stepwise, with monitoring if necessary (23).	2a
Assistive devices	1. The use of pressure-relieving mattresses, wheelchair cushions, and other supportive pads is recommended to create an appropriate support surface. The use of circular air rings should also be avoided. (20, 26).	5b
	2. When needed, mobility aids (e.g., crutches, walkers) should be used to assist with gait and balance impairments, as well as to improve mobility efficiency and safety. The need for gait aids, wheelchairs, and other assistive devices should be assessed on an individual basis (23).	1c
	3. Use of protective positions and postures: use of knee supports, arm supports in the sitting position, and use of a shoulder sling during ambulation can help prevent shoulder subluxation and shoulder pain (20).	1b
	4. Resting hand-wrist splints, along with regular stretching and spasticity management, may be considered in patients with limited active hand movement (26, 30).	1b
	5. Routine use of splints is not recommended (19, 23, 30). The use of splints and taping are not recommended for prevention of wrist and finger spasticity after stroke (26).	1b
	6. Ankle splints used at night and during assisted standing may be considered to prevent ankle contracture in the hemiparetic lower extremity (21, 26).	1c
	7. Ankle-foot orthoses should be used on selected patients with foot drop following proper assessment and with follow-up to verify its effectiveness (19, 23).	1b
	8. In patients with subluxation of the shoulder joint, the use of a rigid shoulder brace is recommended to prevent subluxation progression (20, 24, 26).	1c
	9. The use of splints should be considered on an individualized basis, and a plan for monitoring the effectiveness of splints should be implemented and followed (23, 25)	5b
	10. Continuous cast immobilization or static adjustable splints may be considered to reduce mild to moderate elbow and wrist contractures (26).	5c
Clinical considerations	1. Excessive shoulder flexion, abduction, and pulley-like movements with the hands raised above the head should be avoided, as these movements can lead to uncontrollable shoulder abduction and thus shoulder pain (24).	5b
	2. The arm should not be moved beyond 90 degrees of shoulder flexion or abduction, unless the scapula is upwardly rotated and the humerus is laterally rotated (23, 25).	2a
	3. In the early stage of the disease, it is essential to protect and immobilize the affected arm both at rest and during movement, so as to avoid mechanical injury to the shoulder joint caused by forceful traction (23).	2b
	4. Protecting and supporting the arm during wheelchair use; examples include using a hemi-tray, arm trough, or pillow (23).	5b

## 4 Discussion

### 4.1 Improve team building

Robust evidence demonstrates that organized, multidisciplinary stroke care not only reduces mortality rates and the likelihood of long-term disability but also enhances patient recovery and independence in activities of daily living (26). Clinical practice guidelines specify that core stroke rehabilitation teams should include physiotherapists, physicians with stroke rehabilitation expertise, occupational therapists, speech-language pathologists (SLPs), nurses, social workers, and dietitians, with patients and their caregivers systematically integrated as essential stakeholders in the therapeutic decision-making process (26). Familial support serves as a critical determinant in post-stroke recovery processes, with caregivers typically exhibiting stronger emotional bonds and functioning as primary care providers. Given the protracted trajectory of stroke rehabilitation, family members deliver multidimensional support—spanning emotional sustenance, instrumental assistance, and tangible care—throughout the disease continuum, directly influencing functional outcomes and psychosocial adaptation (31). Therefore, current evidence-based guidelines recommend early and active engagement of both patients and their caregivers in rehabilitation programs, emphasizing the strategic utilization of their self-efficacy to potentiate therapeutic outcomes and optimize health-related quality of life.

### 4.2 Raising awareness of positioning assessment

Positioning management, as a nursing intervention for stroke patients, boasts distinct advantages such as simple operation, low cost, diverse functions, strong clinical applicability, and high safety.

Existing evidence clearly indicates that posture exerts a significant impact on the hemodynamics of stroke patients (32, 33). From a theoretical perspective, different postures can produce differentiated clinical effects: when the supine position (0°) is adopted, the gravitational force helps increase cerebral blood flow in the ischemic penumbra, thereby improving the oxygenation status of brain tissue. This is of positive significance for alleviating neurological damage within the first few hours to days after a stroke. In contrast, the head-of-bed elevation position can effectively reduce intracranial pressure and lower the risk of aspiration pneumonia.

It is important to note that stroke patients are often accompanied by abnormal muscle strength and muscle tone. Therefore, posture management cannot follow a “one-size-fits-all” approach and must be tailored to the specific clinical conditions of individual patients. Hence, before implementing posture management, a comprehensive clinical assessment is required to consider multiple factors, including the type of cerebrovascular disease, physiological indicators such as cerebral blood flow and intracranial pressure, the patient’s own muscle strength and limb motor function level, as well as the presence of relevant comorbidities (28). Consequently, effective positioning management for stroke patients mandates a thorough assessment of the individual’s condition to determine the optimal and safest approach.

### 4.3 Head position

Head position significantly influences cerebral hemodynamics in stroke patients, with the supine position enhancing cerebral perfusion. A meta-analysis shows that in acute ischemic stroke patients, supine head positioning at 0° or 15° significantly increases blood flow velocity in the affected middle cerebral artery (MCA) compared with 30° head elevation and no significant changes in cerebral hemodynamics were observed in the contralateral hemisphere (34). However, whether this perfusion improvement translates to better clinical outcomes remains unclear. Randomized trials indicate that 30° head elevation does not significantly improve 3-month functional outcomes in moderate-to-severe stroke patients compared with the supine position (35). Despite these uncertainties about functional outcomes, current clinical evidence still recommends a supine position (0° head elevation) for cerebral infarction patients to maximize cerebral perfusion pressure (CPP). A multicenter randomized controlled trial found that initiating immediate supine positioning upon hospital admission and maintaining it for at least 24 h improves perfusion outcomes in acute ischemic stroke patients (36). Clinical guidelines also support maintaining a horizontal supine position during the acute phase to optimize CPP (22, 27). However, supine positioning should be temporary: most patients require repositioning after 24–48 h due to the increased risk of aspiration from prolonged flat lying (28).

Elevating the head position is a recommended practice in the management of patients with elevated intracranial pressure (ICP). In contrast to the general recommendation for supine positioning in acute ischemic stroke, elevating the head position is a recommended practice for patients with elevated intracranial pressure (ICP)—though the degree of elevation requires careful consideration, as angles exceeding 45° may reduce CPP. An observational study explored this balance: researchers systematically evaluated the hemodynamic effects of backrest elevation at 15° and 30°, followed by return to the baseline supine position (0°), with continuous monitoring of ICP, mean arterial pressure (MAP), CPP, and peak mean flow velocity in the MCA (37). Elevation to 30° significantly reduced ICP but was accompanied by concomitant decreases in MAP and CPP. Although the supine position achieved maximal CPP, it paradoxically correlated with the highest ICP levels. For patients with large-area cerebral infarction, the supine position may be considered when the risk of cerebral herniation is low and perfusion optimization is prioritized (38). However, prolonged supine positioning must be avoided to mitigate aspiration risk.

In summary, head-of-bed (HOB) positioning requires an individualized approach guided by comprehensive neurological and respiratory assessments, particularly with vigilant monitoring for aspiration risks in patients exhibiting impaired swallowing function or altered consciousness levels (26).

### 4.4 Implementation of positioning management

Positioning management for stroke patients should be individualized through comprehensive assessment to maximize its benefits. Multiple randomized controlled trials (39, 40) have demonstrated that stroke patients receiving enteral nutrition with the head of bed (HOB) elevated ≥30° exhibit significantly lower rates of aspiration, pulmonary infections, and gastric regurgitation compared to those positioned at angles <30°. Furthermore, a systematic review (41) has confirmed that maintaining HOB elevation between 30° and 45° during feeding further reduces the incidence of aspiration-related complications—including pulmonary infections, regurgitation, and abdominal distension—in post-stroke patients with dysphagia. Beyond preventing aspiration, positioning management also plays a critical role in addressing post-stroke spasticity—a common positive symptom following central nervous system injury that may present with dystonic features in stroke patients, characterized by abnormally increased muscle tone and motor impairments (42). Post-stroke spasticity not only compromises a patient’s functional independence but also imposes multiple clinical burdens (43). Of particular clinical relevance is the exacerbation of secondary complications—including shoulder-hand syndrome, pressure injuries, and disuse atrophy—when improper positioning strategies are employed. The anti-spasticity position is a temporary treatment position designed based on the theory of Bobath technology. The main principle is to fight against abnormal movement patterns, control muscle spasm and promote the emergence of separation movement through static reflex inhibition and continuous control (44). The anti-spasticity positioning protocol, a cornerstone intervention in neurorehabilitation, serves as an evidence-based strategy to prevent hemiplegic complications including but not limited to glenohumeral subluxation, shoulder pain, muscle contractures, equinovarus deformity, foot drop, and disuse syndrome. In addition to anti-spasticity positioning, systematic repositioning every 1–2 h achieves pressure redistribution, thereby mitigating tissue injury risks inherent in prolonged immobility. The position content mainly includes the healthy side lying position, the affected side lying position and the bed sitting position. Three therapeutic positions form the protocol’s core implementation framework. The hemiplegic-side lying position is prioritized for its dual capacity to enhance sensory integration in the affected limb while mechanically elongating spastic muscle groups, all without restricting functional use of the non-paretic extremity. In contrast, the healthy-side lying position requires vigilant monitoring to prevent neglect of the hemiplegic limb. Bed-sitting postures, when hemodynamically appropriate, offer advantages in trunk stabilization and nutritional support, provided intracranial hypotension has been conclusively ruled out (24). The post-stroke patient’s position should be placed without affecting the patient’s vital signs. Simultaneously, attention should be paid to protecting the affected limb, avoiding upper limb flexion and excessive extension of the lower limb (25). Regarding the duration of therapeutic positioning, formal guidelines on this parameter for post-stroke patients remain undefined. A meta-analysis (29) has shown no linear correlation between positioning duration and functional outcomes. Current evidence recommends a minimum intervention duration of 4 weeks, with specific timelines determined through individualized patient assessment.

### 4.5 Preventing complications

Proper positioning management can prevent many post-stroke complications. Pressure injury development is significantly correlated with patient positioning (45). In stroke patients, hemiplegia, sensory alterations, and consciousness level changes predispose individuals to risks of joint/muscle contractures and cutaneous breakdown (46). To address this, clinical guidelines emphasize minimizing friction, redistributing pressure via appropriate support surfaces, and controlling moisture.

Hemiplegic shoulder pain (HSP) is one of the common complications after stroke, with a prevalence of 22% to 47% (47), often causes moderate-to-severe pain that disrupts upper limb rehabilitation, delays functional recovery, and prolongs hospitalization. Shoulder-hand syndrome represents a specific subtype of shoulder pain, frequently complicating shoulder subluxation, where the primary management goal is to prevent progression. Numerous guidelines highlight positioning intervention as a key strategy in managing spasticity, shoulder pain, and shoulder-hand syndrome. Protective postures can mitigate the risk of shoulder pain and subluxation (20). For patients with existing post-stroke shoulder pain, positioning management should prioritize maintaining scapulohumeral symmetry, typically positioning the shoulder in 30° abduction, 15°external rotation, and 20° forward flexion. If shoulder pain or subluxation already occurs, positioning adjustments then focus on preventing further deterioration. Positioning devices such as arm rests and supportive slots can further aid this by providing stable support and maintaining proper joint alignment, thereby minimizing discomfort and subluxation (26). However, it is critical to avoid improper positioning practices. For example, pulley-like movements that lift the affected hand high above the head can cause excessive shoulder flexion and abduction, damaging local joint capsules and ligaments and exacerbating shoulder pain. Such inappropriate movements not only worsen existing shoulder injuries but also hinder patients’ active rehabilitation efforts (24).

### 4.6 Auxiliary appliances

An orthotic is an externally applied device designed to restore anatomical alignment, maintain functional positioning, and assist bodily function (48). The Chinese Stroke Nursing Guidelines explicitly recommend selecting hand, wrist, ankle, and foot orthoses as needed during post-stroke rehabilitation to prevent complications, with careful consideration of device appropriateness emphasized (25).

Spasticity is common in the upper extremities, most commonly in the elbow (79%), wrist (66%), and shoulder (58%) (6). Spasticity-related contractures not only cause pain but also impair self-care abilities, including dressing and personal hygiene. Static stretching is a widely used type of stretching that can be applied in a variety of ways, including the physical therapist’s hands, splints, orthotics, and cast models (49). For stroke patients specifically, orthotics aim to reduce spasticity, enhance function, prevent contractures, alleviate pain, and decrease swelling. Wrist and hand orthoses stabilize the limb in a functional position, serving as effective passive stretching tools to target wrist flexor spasticity. Meta-analyses indicate that orthotic interventions typically span 3–4 weeks, applied 6–7 days weekly for at least 20 min daily (49). However, while guidelines (23, 26) recommend hand splints to prevent wrist and finger contractures, their efficacy in reducing wrist spasticity remains controversial (50). In light of this uncertainty, current clinical guidelines advises cautious use: short-term splinting may prevent contractures, but long-term or routine use is generally discouraged (23). Thus, individualized splinting plans with regular efficacy monitoring are essential (23, 30).

Shoulder stabilization devices, including orthoses and slings recommended in guidelines, provide mechanical support to reduce early subluxation and late contracture. These devices maintain shoulder joint anatomy by stabilizing muscles and bones, thereby relieving pain and preventing/correcting subluxation. Proximal-distal orthopedic appliances for the affected arm have been shown to improve shoulder pain and reduce subluxation (51), while shoulder straps alleviate discomfort by immobilizing the limb (52). Lower limb orthoses are one of the earliest and most widely used orthoses in history. They support weight, prevent and correct lower limb deformities, effectively compensate for the function of paralyzed muscles, limit unwanted movement of lower limb joints, and help treat lower limb motor dysfunction by improving posture when standing and walking (53). Currently, hemiplegic patients commonly use various lower limb orthoses, which are categorized by the limb segment they target: knee orthoses, ankle orthoses, knee-ankle-foot orthoses, and hip-knee-ankle-foot orthoses. Of these, ankle-foot orthoses (AFOs) are the most frequently used. Beyond addressing motor function, such devices can also improve balance—for example, canes or AFOs. Ankle plantarflexion contracture after stroke, for instance, can compromise gait quality and safety; guidelines therefore recommend AFOs for patients with remediable gait disorders to compensate for foot drop, improve walking stability, and potentially prevent ankle contractures (26, 54).

Orthotic use must be guided by comprehensive patient assessment, incorporating factors such as spasticity severity, functional deficits, and comorbidities (e.g., intracranial pressure dynamics). While orthotics offer evidence-based benefits in preventing contractures and improving mobility, their application should always be individualized, with routine evaluation of efficacy and adjustment of intervention duration.

## 5 Limitations

This study aimed to summarize the best evidence for positioning management in patients with stroke. However, it has several limitations. First, the literature included in this study was restricted to Chinese and English, which may have led to the omission of evidence from other languages and thus compromised the comprehensiveness of the data. Second, the quality assessment of different types of literature using various tools may not be fully consistent, and the integration of conflicting or similar evidence may lack sufficient precision for practical application. Third, the evidence in this study was derived from different countries, where there are still certain differences in social status, clinical settings, and cultural backgrounds.

## 6 Conclusion

Positioning management of stroke usually involves several interdependent aspects. Scientific and standardized postural management is critical for preserving limb function and preventing complications in stroke patients. This study systematically screened and synthesized the best available evidence on postural management for stroke patients, categorizing it into seven domains: team composition, comprehensive assessment, head-of-bed elevation angle, body positioning strategies, early mobilization, assistive devices, and clinical considerations. A total of 37 pieces of best evidence were identified, providing an evidence-based framework for healthcare professionals to implement postural interventions. Therefore, it is recommended that future researchers conduct and implement relevant studies based on their countries’ actual conditions to further enrich the evidence and intervention strategies for posture management in stroke patients. On this basis, efforts should be made to promote the formulation of relevant guidelines, provide clear rules for positioning management, and ultimately ensure tangible benefits for patients.
